# Supplementary material for: Chalcone T4 Inhibits RANKL-Induced Osteoclastogenesis and Stimulates Osteogenesis In Vitro
Source: Int J Mol Sci. 2023 Apr 21;24(8):7624. doi: 10.3390/ijms24087624 (PMC10141037; doi:10.3390/ijms24087624)

Supplementary Table S1: Primers used for RT-PCR.

Supplementary Figure S1: Chemical structure of Chalcone T4.

Supplementary Figure S2: Change in the viability of the RAW 264.7 cells compared to the control after treatment with different concentrations of Chalcone T4 (2.5, 5, 10 and 20 μM) for 6 days. Cells treated with camptothecin (CPT) (apoptosis-inducing compound) were used as a positive control. Only the dose of 20 μM of chalcone T4 reduced the cell viability in relation to the control, but the difference was not statistically significant. (*) $p<0.05$ compared with control group. The bars indicate the percentage of cell viability (%) and vertical lines the standard error of the mean (SEM) from three different experiments assessed in triplicate.

Supplementary Figure S3: Viability of the MC3T3 cells treated with different concentrations of Chalcone T4 or camptothecin (CPT) for 7 days. None of the concentrations of the compound altered the viability of the cells when compared to the control.

Supplementary Table S1

Gene	Accession #	Amplicon (bp)
Oscar	NM_001290377	72
Trap	NM_001102404	79
Mmp-9	NM_013599	76
Ctsk	NM_031560	69
Runx-2	NM_001145920	91
Alp	NM_007431.3	60
Gapdh	NM_017008	87

Supplementary Figure S1

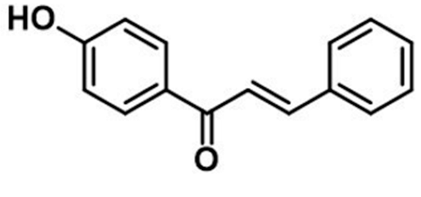

(MW) = 224,25 g/mol

Supplementary Figure S2

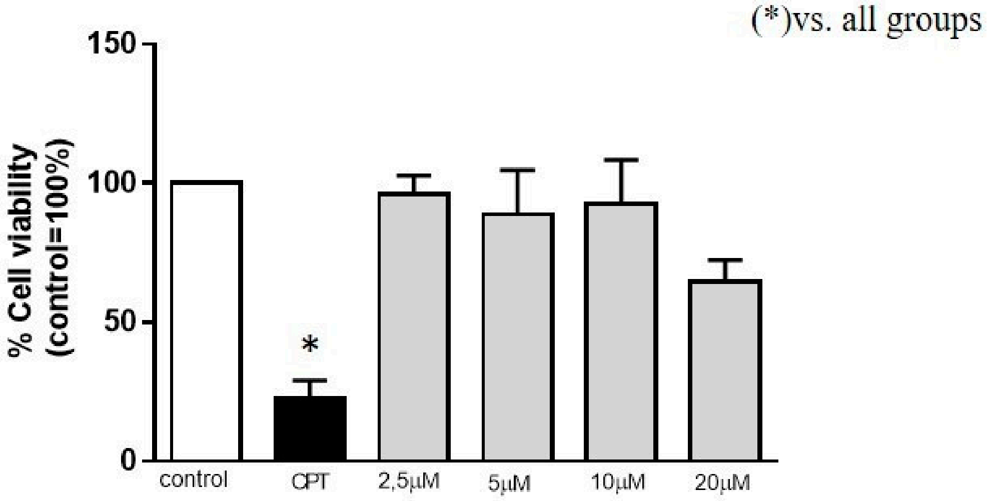

Supplementary Figure S3

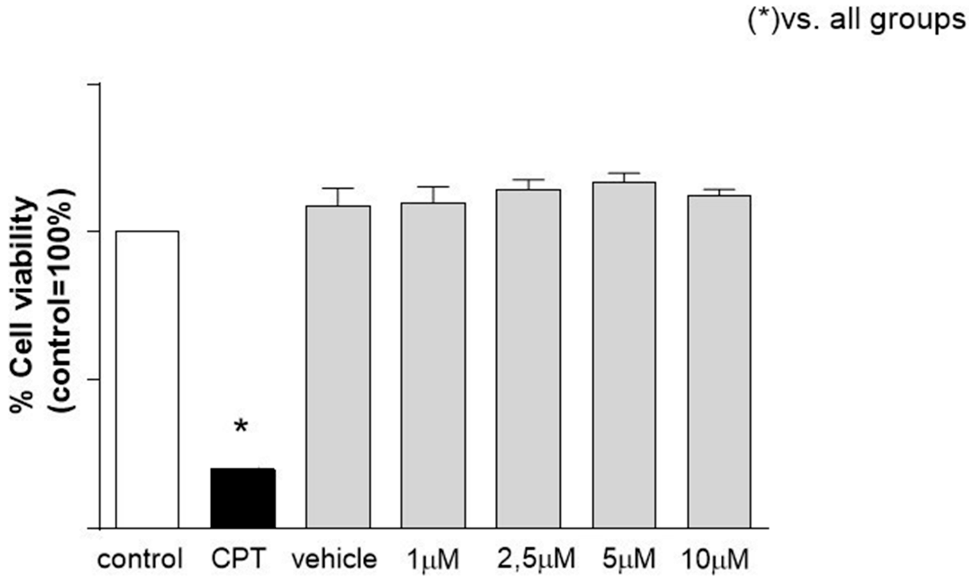

Supplement: Supplementary file 1 [file ijms-24-07624-s001.zip › ijms-2305709-supplementary.pdf]
